# Blast Exposure Impairs Sensory Gating: Evidence from Measures of Acoustic Startle and Auditory Event-Related Potentials

**DOI:** 10.1089/neu.2018.5801

**Published:** 2019-02-09

**Authors:** Melissa A. Papesh, Jonathan E. Elliott, Megan L. Callahan, Daniel Storzbach, Miranda M. Lim, Frederick J. Gallun

**Affiliations:** ^1^ National Center for Rehabilitative Auditory Research, VA Portland Health Care System, Portland, Oregon.; ^2^ Department of Otolaryngology Head and Neck Surgery, Oregon Health & Science University, Portland, Oregon.; ^3^ Department of Neurology, Oregon Health & Science University, Portland, Oregon.; ^4^ Department of Psychiatry, Oregon Health & Science University, Portland, Oregon.; ^5^ Department of Medicine, Division of Pulmonary and Critical Care Medicine, Oregon Health & Science University, Portland, Oregon.; ^6^ Department of Behavioral Neuroscience and Oregon Institute of Occupational Health Sciences, Oregon Health & Science University, Portland, Oregon.

**Keywords:** electrophysiology, hearing, post-traumatic stress, TBI, veteran

## Abstract

Many military service members and veterans who have been exposed to high-intensity blast waves experience traumatic brain injury (TBI), resulting in chronic auditory deficits despite normal hearing sensitivity. The current study sought to examine the neurological cause of this chronic dysfunction by testing the hypothesis that blast exposure leads to impaired filtering of sensory information at brainstem and early cortical levels. Groups of blast-exposed and non-blast-exposed participants completed self-report measures of auditory and neurobehavioral status, auditory perceptual tasks involving degraded and competing speech stimuli, and physiological measures of sensory gating, including pre-pulse inhibition and habituation of the acoustic startle reflex and electrophysiological assessment of a paired-click sensory gating paradigm. Blast-exposed participants showed significantly reduced habituation to acoustic startle stimuli and impaired filtering of redundant sensory information at the level the auditory cortex. Multiple linear regression analyses revealed that poorer sensory gating at the cortical level was primarily influenced by a diagnosis of TBI, whereas reduced habituation was primarily influenced by a diagnosis of post-traumatic stress disorder. A statistical model was created including cortical sensory gating and habituation to acoustic startle, which strongly predicted performance on a degraded speech task. These results support the hypothesis that blast exposure impairs central auditory processing via impairment of neural mechanisms underlying habituation and sensory gating.

## Introduction

The Department of Defense reports that between the years 2000 and 2017, nearly 380,000 military service members have been diagnosed with a traumatic brain injury (TBI), with >312,000 of these classified as mild (mTBI).^1^ The majority of these cases stemmed from exposure to high-intensity blast discharges, with incidence of blast exposure among military service personnel reaching levels as high as 8.3% in recent conflicts.^2^ Of those with deployment-related TBI, cognitive and sensory impairments are among the most prevalent symptoms reported,^3,4^ and these often persist for months or years following trauma.^5–8^

Among the most common sensory impairments reported following TBI are auditory complaints. Studies indicate that between 58% and 87% of veterans with mTBI stemming from blast exposure report auditory difficulties.^9–11^ The most common auditory complaints among these veterans include increased sensitivity to noise, poor speech understanding in the setting of competing background noise, difficulty understanding rapid speech, problems understanding speech on the telephone, difficulty following conversations among groups of people, poor recall of auditory information, and auditory fatigue.^5,6,9,12–16^ Hearing problems often persist in spite of normal pure-tone hearing sensitivity, suggesting a central rather than peripheral locus of dysfunction. This assumption is corroborated by recent work showing that between 20% and 40% of veterans exposed to high-intensity blasts demonstrate abnormal performance on behavioral and electrophysiological assessments of the central auditory system.^17,18^ Similar patterns of auditory deficits have been reported in blast-exposed veterans even after several years of recovery after blast exposure.^6^

One potential mechanism by which blast exposure affects auditory processing could be impairment of “sensory gating,” or the process by which sensory information is filtered for relevant content prior to being processed by higher order cognitive regions. Sensory gating occurs at pre-attentive levels of stimulus processing and plays an important role in preventing interference by sensory stimuli during higher-order functions such as attention and memory.^19,20^ Reduced sensory gating, measured using a paired-click auditory evoked potentials (AERP) paradigm, was previously reported in a study of TBI patients with attention and memory dysfunction,^21^ providing support for a potential link between blast exposure and impaired sensory gating. Poor sensory gating could account for auditory-specific deficits in blast-exposed veterans such as difficulty understanding speech signals in the context of background noise and comprehension of rapidly spoken speech, as well as cognitive symptoms such as difficulty attending to and recalling long conversations and lists of spoken instructions.

Common methods of assessing sensory gating include: (1) measurement of habituation^22,23^ and pre-pulse inhibition (PPI)^24^ of the acoustic startle reflex (ASR), which primarily evaluates brainstem pathways; and (2) AERP elicited using a well-established paired-click paradigm^25–27^ that primarily targets responses from thalamocortical and cortical pathways.

Therefore, in the current study, we aimed to explore the relationship between auditory perception (using self-report and behavioral testing) and sensory gating using both acoustic startle and AERP in veterans with previous exposure to high-intensity blast waves. We hypothesized that blast-exposed veterans would demonstrate reduced sensory gating and that poor auditory perception in blast-exposed veterans would be correlated with biological markers (i.e., habituation to ASR and AERP) of impaired sensory gating.

## Methods

### Participants

Twenty-nine combat-deployed veterans were recruited from the Veterans Affairs Portland Health Care System (VAPORHCS) in Portland, Oregon, including those who had been exposed to high-intensity blast discharges within the past 10 years, as well as those with no history of blast exposure or brain injury. Potential participants completed an interview regarding their military service, prior head trauma, and blast exposure(s), including symptoms immediately and within the first 24 h of injury. Only participants who met basic criteria for brain injury (e.g., disorientation, ringing in the ears, nausea, light sensitivity, loss of consciousness, post-traumatic amnesia, and headache immediately following the event) established by the Defense and Veterans Brain Injury Center (DVBIC) were admitted to the blast-exposed participant group. All participant reports were consistent with a classification of mTBI. Although a medical chart review was also performed to determine if a clinical diagnosis of TBI had been made, blast-exposed participants without the clinical diagnosis of TBI were still included in the study, as previous work has indicated that patients who meet the DVBIC criteria for brain injury following blast exposure experience auditory dysfunction similarly to those who have received a blast-related diagnosis of TBI.^6,17,18,28^ Additional exclusionary criteria included pure-tone hearing thresholds >30 dB hearing level (HL) at any test frequencies between 250 and 4000 Hz, threshold differences of >10 dB between the left and right ear at any test frequency, and diagnosis of mental health conditions associated with poor sensory gating including schizophrenia, bipolar disorder, obsessive compulsive disorder, and attention-deficit/hyperactivity disorder. Sixteen participants (all males) were admitted to the blast-exposed group (average age 36.9 years; age range 24–58 years), and 13 participants (2 females) were admitted to the control group (average age 38 years, age range 19–66 years). The Institutional Review Board at the VAPORHCS approved this project, and all subjects provided oral and written informed consent prior to participation.

### Study overview

**FIG. 1.**
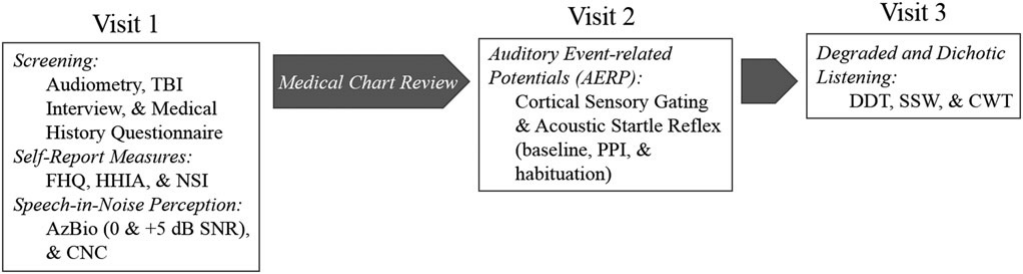
Schematic representation of study visits and procedures.

Testing was completed over two to three visits (Fig. 1). Visit 1 consisted of screening measures, including an audiological assessment, an interview of past head traumas, and a medical history questionnaire. Once inclusionary criteria were confirmed, participants then completed speech-in-noise perception testing as well as additional self-report questionnaires. Following visit 1, a medical record review was conducted to determine if a diagnosis of post-traumatic stress disorder (PTSD) was present, and to confirm self-reported reported medical history. Visit 2 consisted of sensory gating testing, including both the ASR and AERP analyses. All participants completed visits 1 and 2.

Visit 3, which was not part of the original study design, was added to further assess comprehension of competing and degraded speech. All participants were invited back for this third visit; 15/16 blast-exposed participants, and 9/13 control participants returned for this test session.

### Self-report measures

All participants completed a medical case history questionnaire including questions assessing audiological history, military service, other health diagnoses, and use of medications, cigarettes, alcohol, and illicit drugs. The Functional Hearing Questionnaire (FHQ), developed for veterans with brain injuries was used to evaluate self-perceived hearing difficulties.^5^ The FHQ is a nine item questionnaire that asks participants to rate their level of difficulty hearing in different circumstances on a four point scale. Scores range from 9 to 36, with higher scores indicating a greater level of difficulty. The Hearing Handicap Inventory for Adults (HHIA)^29^ was used to assess the social and emotional impact of hearing difficulties on everyday life. The HHIA is a 25 item questionnaire that asks patients to rank how often auditory issues create problems in daily life. Scores range from 0 to 100, with higher scores indicating greater perceived levels of handicap. Lastly, all participants completed the Neurobehavioral Symptom Inventory-22 (NSI)^30^ to assess post-concussive symptom severity. Each item is scored from 0, indicating no problem, to 4, indicating a severe debilitating problem.

### Behavioral speech perception testing

All speech testing was completed in a sound-attenuating chamber using ER3-A insert earphones (Etymotic Research, Elk Grove Village, IL). Speech-in-noise tests were selected from the Minimum Speech Test Battery^31^ and presented via a CD player routed through an audiometer. The AzBio was selected as a sentence-level speech perception test. This test is composed of target sentences spoken by two male and two female speakers using conversational speech patterns presented at a level of 60 dB HL.^32^ Conditions included binaural presentation of target sentences in quiet, and in the presence of 10-talker speech babble presented at 55 dB HL and 60 dB HL resulting in signal-to-noise ratios (SNR) of +5 dB and 0 dB for the two speech-in-babble conditions, respectively. Each condition included 20 target sentences that participants were asked to repeat to the researcher. Word-level speech perception was measured using the Consonant-Nucleus-Consonant (CNC) test.^33^ CNC test materials are comprised of monosyllabic words spoken by a female talker, each preceded by the carrier phrase “Ready”. Each word consists of three speech phonemes including two consonant sounds with a vowel in the middle. CNC target words were presented at a level of 60 dB HL in quiet and in 55 dB HL of background noise (+5 dB SNR) with a spectrum matching the average long-term spectrum of speech. In each condition (quiet and noise), responses were scored based upon the number of phonemes correctly repeated with a maximum score of 150 for each condition. Results for both the AzBio and CNC tests are presented as percent correct for each condition.

Additional speech tests included in the third test session consisted of two tests of competing dichotic speech (the Dichotic Digits Test [DDT] and the Staggered Spondaic Words [SSW] test) and one test of degraded speech (the Compressed Word Test [CWT] test). Each of these tests were developed for the clinical assessment of central auditory processing disorders, and previous research has indicated that blast-exposed veterans often perform poorly on these measures.^6,18^ Tests were presented at a level of 35 dB above each participant's speech recognition threshold, determined during the audiological evaluation at the first visit, to ensure equivalent levels of audibility across all participants. The DDT consists of four numbers, two spoken to each ear nearly simultaneously.^34^ Participants were asked to repeat back all four numbers in any order, and scores were reported as percent correct. The SSW test consisted of 40 pairs of “spondaic” words, each “spondee” consisting of two complete one-syllable words (e.g., “cupcake”) spoken with equal emphasis on both syllables.^35^ In each trial, the two spondees were presented, one to each ear, such that the first syllable of the first spondee was presented in quiet in one ear and the second syllable of the first spondee overlapped the first syllable of the second spondee in the opposite ear. Participants were asked to repeat back both words and scores were reported as the total number of errors out of all 80 words. Lastly, the CWT test consisted of 50 monosyllabic words time-compressed to 45% of their original duration.^36^ Each ear was tested separately and participants were asked to repeat back each word, with scoring reported as percentage of words correctly repeated.

### Measurement of PPI and habituation of the ASR

The acoustic startle stimulus consisted of a 40 ms duration burst of white noise presented at a level of 113 A-weighted decibels (dBA) with a nearly instantaneous rise time. Stimuli were presented binaurally via ER3A insert earphones (Etymotic, Elk Grove Village, IL) over a continuous background of white noise presented at 60 dB A. All stimulus presentations and responses were recorded using the Neuroscan Synamps System II (NeuroScan Inc., El Paso, TX). The skin below the left eye and on the forehead was first exfoliated and then cleansed using an alcohol swab. Eye blink responses to startle stimuli were measured by recording the electromyographic (EMG) activity from the orbicularis oculi muscle under the left eye using two EL254 Ag-AgCl electrodes (BIOPAC Systems, Inc., Goleta, CA) spaced ∼25 mm apart. An additional ground electrode was placed on the forehead.

Participants were seated in a reclining armchair within a double-walled sound-attenuating chamber and instructed to look at a static image of a beach scene located on the wall in front of them to maintain vigilance and eye gaze. Prior to testing, all participants were given the opportunity to listen to the acoustic startle stimulus to ensure tolerability. Testing began with the background white noise presented for 2 min to allow participants to acclimate to the sound and test environment. All stimuli were presented at an average inter-trial interval of ∼45 sec (range 35–55 sec). Four startle stimuli were initially presented, and the average EMG magnitude in response to these four initial startle stimuli was taken as the baseline startle response. Participants were then presented with 16 additional startle stimuli, 8 of which were preceded by a low-amplitude pre-pulse tone presented 120 ms before the startle stimulus. The sequence of startle-only and prepulse and startle stimuli were randomized for each participant. The experiment ended with four additional startle stimuli (without pre-pulse tones), and the average EMG response to these final four stimuli was taken as the habituated startle response.

The raw EMG signal was amplified, sampled at 1000 Hz, bandpass filtered between 30 and 200 Hz, and rectified. Responses to startle stimuli were defined as EMG peak amplitude in a time window from 20 to 120 ms after stimulus presentation. Trials with excessive EMG artifact, which obscured the response stimulus, were excluded (∼3%, or 12 of the 416 total trials). PPI was defined as the reduction in the magnitude of response from startle stimulus-only trials to prepulse and startle stimulus trials, and was calculated as the percent change in amplitude. Habituation was defined as the reduction in response magnitude from the initial four startle stimulus presentations to the final four startle stimulus presentations, and was calculated as the percent change in amplitude.

### Measurement of cortical sensory gating

All participants were asked to abstain from consuming caffeine and smoking cigarettes for a minimum of 2 h prior to completing the cortical sensory gating protocol, to avoid excitation of nicotinic receptors, which can temporarily improve sensory gating.^37,38^ Participants were seated comfortably in a sound attenuating booth, and instructed to ignore the test stimuli and watch a silent closed-captioned movie of their choice. A validated paired-click testing paradigm was used,^25–27^ to target thalamocortical and auditory cortex responses. On each trial, two identical 10 ms clicks were presented with an inter-stimulus interval of 500 ms. The conditioning (first click) and test (second click) stimuli were presented binaurally at a level of 80 dB C via ER3A insert earphones. A total of 200 trials (consisting of both a conditioning and a test click) were presented. ERP responses were obtained using a 64 channel cap (Electro-Cap International, Inc.; Eaton, OH) and the Compumedics Neuroscan System (Charlotte, NC). The ground electrode was located on the forehead, and Cz served as the reference electrode during recordings. Data were then re-referenced off line to an average reference of all electrodes. Horizontal and vertical eye movement was monitored with electrodes located inferiorly and at the outer canthi of both eyes. The recording time window consisted of a 200 ms pre-stimulus baseline following by an 1100 ms post-stimulus onset. Responses were analog low-pass filtered online at 100 Hz (12 dB/octave roll off), and all channels were amplified with a gain × 10 and converted using an analog-to-digital sampling rate of 1000 Hz. Trials with eye blink artifacts were corrected off line using Neuroscan software. This blink reduction procedure calculates the amount of covariation between each active recording channel and a vertical eye channel using a spatial, singular value decomposition, and removes the vertical blink activity from each electrode on a point-by-point basis to the degree that the evoked response and blink activity co-varied. After blink correction, trials containing artifacts > ±70 μV were rejected from averaging. For all individuals and conditions, ≥70% of the collected trials were available for averaging after artifact rejection. After artifact rejection, the remaining sweeps were averaged (an average of 178 sweeps per participant after artifact rejection and a minimum of 142 sweeps) and filtered off line.

The latencies and amplitudes of P1, N1, and P2 peaks were obtained from the central electrode Cz. For N1 and P2 peaks, offline bandpass filters from 0.1 Hz (high-pass filter, 24 dB/octave) to 30 Hz (low-pass filter, 12 dB/octave) were used to maximize cortical responses while reducing extraneous noise. P50 peaks were analyzed after applying a bandpass filter from 10 to 30 Hz, because of the higher frequency content of thalamocortical responses.^39^ Peak values were confirmed by comparing peaks at the Cz electrode location with peaks in the inverted waveforms measured at temporal electrode locations, as well as comparison with global field power traces for each individual, and grand averaged subject group responses. Amplitude values were measured relative to pre-stimulus baseline. Gating was defined as the reduction in peak amplitude from the conditioning (S1) to the test stimulus (S2) of a stimulus pair, and calculated as percent change in response amplitude [(S1amplitude-S2amplitude/S1amplitude) × 100]. The change in amplitude between responses to conditioning and test stimuli is hereafter referred to as ΔP1, ΔN1, and ΔP2, to denote changes in each peak.

### Statistical analysis

All statistical analysis was completed using SPSS. Group differences for all test measures were assessed via one way ANOVA with α set *a priori* to *p* < 0.05. Bivariate correlations were conducted using Kendall's tau-b to assess relationships among self-report, demographic, behavioral, and physiological test measures. Kendall's tau was used because it is independent of underlying distributions and because the resulting significance values are more accurate in small sample sizes than those of other non-parametric measures such as Spearman's rho.^40,41^ In light of the multiple comparisons necessary in the correlation analysis, we employed a Benjamani–Hochberg correction to limit the false discovery rate to ≤10%. Last, stepwise multiple linear regression was used to determine which test variables were significant predictors of sensory gating indices and behavioral test performance.

## Results

### Participant characteristics

Audiometric thresholds were within the clinically normal range of hearing for both groups, specified as thresholds of ≤25 dB HL at test frequencies between 250 and 8000 Hz. Blast-exposed participants had, on average, significantly more years of military experience, with an average of 11.6 years, compared with controls who had an average of 5.8 years (*F*_(1,27)_ = 4.367; *p* = 0.046). Both groups had high rates of comorbid medical diagnoses, including sleep disorders, depression, anxiety, tinnitus, and migraines. Medical chart review indicated that 13/16 blast-exposed and 5/13 control participants had a diagnosis of PTSD (*F*_(1,27)_ = 20.343; *p* < 0.001). Blast-exposed participants reported an average of 9.75 blast exposures or other types of brain injuries (range from 1 to 30) with an average of 7.1 blast exposures/participant (range from 1 to 30) and the most severe blast injury occurring an average of 7.4 years previous to participation in the study (range of 3–10 years). Eleven of the 16 blast-exposed participants had a medical chart confirmed diagnosis of TBI, all of which were classified as mild.

### Self-report measures

The upper portion of Table 1 shows the group average and standard deviations of each self-report test measure as well as ANOVA results. Group differences were significant for all self-report measures, with blast-exposed participants endorsing higher perceived levels of auditory dysfunction and handicap on the FHQ and the HHIA, and greater severity of neurobehavioral post-concussive symptoms on the NSI compared to control group participants. Overall, group differences of perceived auditory function on the FHQ were driven by greater difficulty understanding on the telephone (*F*_(1,27)_ = 6.458; *p* = 0.017) and when speech was spoken rapidly (*F*_(1,27)_ = 5.649; *p* = 0.025) among the blast-exposed group. Difficulties with understanding in background noise (*F*_(1,27)_ = 3.817; *p* = 0.061) and following long conversations (*F*_(1,27)_ = 4.180; *p* = 0.051) were also endorsed at higher rates among blast-exposed veterans.

**Table 1.** Group Averages, Standard Deviations, and Statistical Analysis for Each Self-Report and Behavioral Test Measure

			*ANOVA*	
*Self-report measures*	*Blast-exposed mean (SD)*	*Control mean (SD)*	F	p
FHQ Total	19.9 (6.1)	15.2 (3.9)	*F*(1,20) = 5.614	0.025
HHIA	32.5 (31.33)	6.75 (12.8)	*F*(1,27) = 4.903	0.037
NSI Total	33.9 (21.3)	14.1 (12.4)	*F*(1,27) = 8.771	0.006

*Behavioral measures*				
AzBio Quiet	99 (1.4)	99.5 (.007)	*F*(1,27) = 1.690	0.205
AzBio +5 dB SNR	94 (.05)	96.6 (.02)	*F*(1,27) = 2.612	0.118
AzBio 0 dB SNR	65 (11.5)	65.4 (10.5)	*F*(1,27) = 0.012	0.913
CNC Quiet	99.8 (.005)	99.9 (.004)	*F*(1,27) = 0.167	0.686
CNC +5 dB SNR	86.5 (6.9)	89.6 (3)	*F*(1,27) = 2.321	0.272
DDT	94.6 (5.3)	97.5 (2.2)	*F*(1,22) = 2.138	0.158
SSW	6.3 (5.9)	2.5 (1.9)	*F*(1,22) = 3.070	0.094
CWT - right	93.7 (6.3)	99 (1.7)	*F*(1,22) = 3.998	0.06
CWT - left	93.3 (4.0)	98 (1.3)	*F*(1,22) = 5.901	0.025

FHQ, Functional Hearing Questionnaire; HHIA, Hearing Handicap Inventory for Adults; NSI, Neurobehavioral Symptom Inventory; SNR, signal-to-noise ratios; CNC, Consonant-Nucleus-Consonant; DDT, Dichotic Digits Test; SSW, Staggered Spondaic Words; CWT, Compressed Word Test.

### Auditory perception measures

**FIG. 2.**
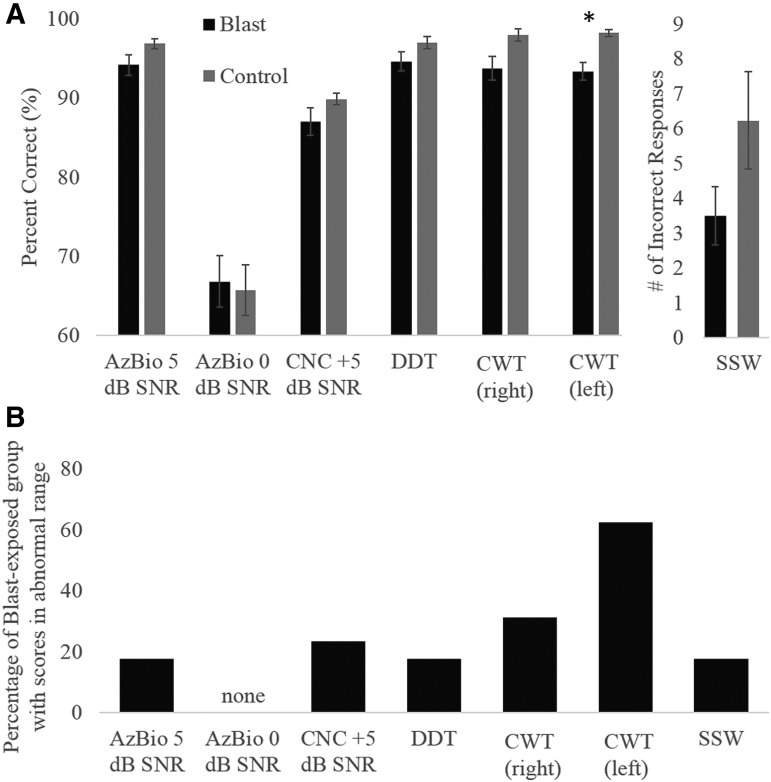
**(A)** Average performance of blast-exposed (black) and control (gray) subjects on behavioral tests of auditory perception. *Indicates significant group difference at the level of *p* < 0.05. Error bars indicate ±1 SEM. **(B)** The percentage of participants in the blast-exposed group with abnormal scores on each of the behavioral auditory perceptual tests. Abnormal performance was defined as >2 standard deviations below the mean performance of control participants.

Average performance of each participant group on all tests of auditory perception are shown in Figure 2A. Performance among the blast-exposed participants tended to be somewhat poorer than that of control participants; however, this difference was only significant for the CWT presented to the left ear (Table 1). Responses to right ear presentations of the CWT (*p* = 0.06) and responses to the SSW (*p* = 0.094) also demonstrated a trend toward significantly worse performance in the blast-exposed group. Based upon the performance criterion of ±2 standard deviations from the mean performance of the control group, 60% of the blast-exposed group scored in the abnormal range on the left-ear presentations of the CWT, whereas 33% scored in the abnormal range in response to right-ear presentations of the CWT (Fig. 2B).

### PPI and habituation of the ASR

**FIG. 3.**
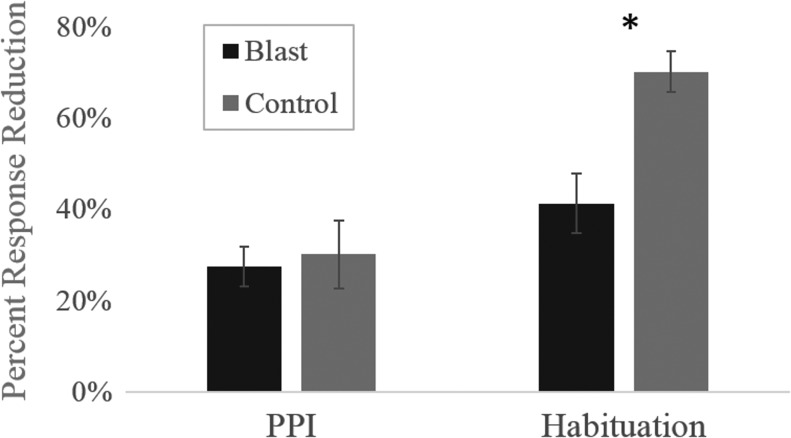
Average percent change in the magnitude of the startle response in blast-exposed (black) and control (gray) participants in response to pre-pulse inhibition (PPI) trials and after habituation to the startle stimulus. *Indicates significant group difference at the level of *p* < 0.05. Error bars indicate ±1 SEM.

Three control and six blast-exposed participants, all with a diagnosis of PTSD, opted not to complete the ASR protocol. Average group effects of PPI and habituation to the startle stimulus for both groups are shown in Figure 3. No significant group differences were found for the magnitude of the baseline ASR (*F*_(1,18)_ = 0.664; *p* = 0.426) or PPI of the ASR (*F*_(1,18)_ = 0.099; *p* = 0.757); however, members of the blast-exposed group exhibited significantly less habituation to repetitions of the acoustic startle stimulus than did the control group (*F*_(1,18)_ = 14.593; *p* = 0.001). Specifically, the ASR magnitude of the control group diminished by 69.5%, compared with the blast-exposed group, which exhibited a reduction of 39%.

### Subcortical and cortical sensory gating

**FIG. 4.**
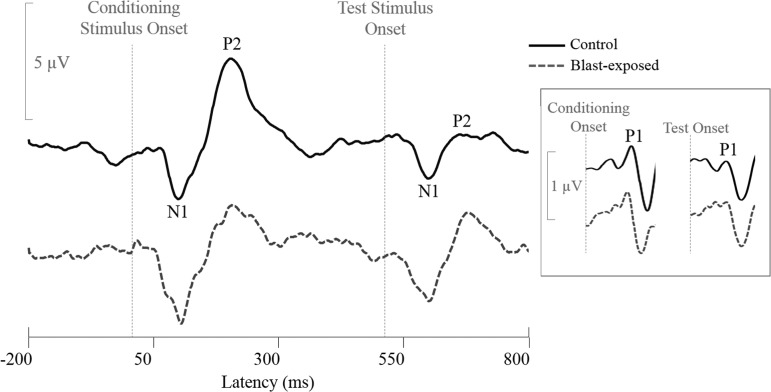
Auditory evoked potentials (AERP) waveforms obtained in a representative control (solid line) and a representative blast-exposed (broken line) participant in response to the paired-click sensory gating paradigm. N1 and P2 responses to both the conditioning and test stimuli are indicated on the control waveform. Inset to the right shows P1 responses, which were analyzed using different filter settings from those of the N1 and P2 peaks.

Analysis of AERP responses to the dual-click paradigm revealed significantly reduced sensory gating in the P2 peak response of the blast-exposed participant group compared with the control group (Table 2). This effect was driven by significantly larger P2 peaks in response to the test stimulus among blast-exposed participants compared with control participants, whereas P2 amplitudes in response to the conditioning stimulus were similar between the two groups. This pattern can be seen in Figure 4, which depicts representative responses of a control (solid black line) and a blast-exposed (broken blue line) participant in response to the AERP sensory gating test. No significant group differences were found in P1 or N1 amplitude in response to the conditioning stimulus, test stimulus, or overall amount of sensory gating for these peaks (Table 2).

**Table 2.** Group Averages, Standard Deviations, and Statistical Group Comparison of Responses to Conditioning and Test Stimuli Presented in the Paired-Click Sensory Gating Paradigm

	*Conditioning (μV) mean (SD)*	*Test (μV) mean (SD)*	*Percent change (%) mean (SD)*
P1			
Blast	0.400 (.36)	0.187 (.26)	54.13 (56.7)
Control	0.606 (.30)	0.262 (.16)	51.68 (20.5)
ANOVA:	*F(*1,27) = 2.713; *p* = 0.111	*F*(1,27) = 0.808; *p* = 0.368	*F*(1,27) = 0.022; *p* = 0.884
N1			
Blast	−2.525 (1.48)	−1.546 (.86)	36.03 (30.7)
Control	−2.775 (2.06)	−1.69 (.98)	30.88 (29.6)
ANOVA:	*F*(1,27) = 0.144; *p* = 0.707	*F*(1,27) = 0.169; *p* = 0.684	*F*(1,27) = 0.209; *p* = 0.651
P2			
Blast	4.722 (2.37)	2.070 (.78)	51.87 (16)
Control	4.213 (1.25)	1.456 (.71)	65.74 (13.8)
ANOVA:	*F*(1,27) = 0.487; *p* = 0.491	***F*(1,27) = 4.783;*p* = 0.038**	***F*(1,27) = 6.085;*p* = 0.020**

Bolded values indicate significance at the level of *p* < 0.05.

### Correlations

**FIG. 5.**
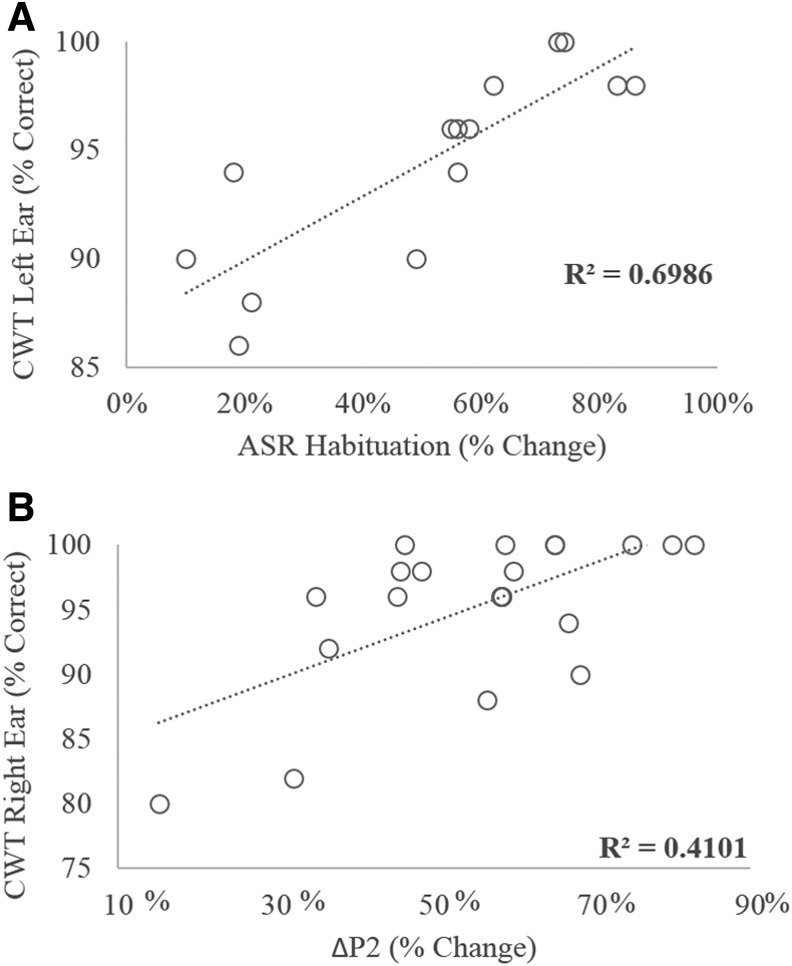
Significant correlations were found between habituation to the acoustic startle reflex (ASR) stimulus and performance on the Compressed Word Test (CWT) presented to the left ear (panel A) and between P2 sensory gating and performance on the CWT presented to the right ear.

To evaluate the relationship between neurophysiology and behavior, correlations were assessed among all measures of sensory gating (PPI, ASR habituation, and ΔP1, ΔN1, and ΔP2) and behavioral measures of degraded and competing speech (AzBio in both noise conditions, CNC in +5 dB SNR, CWT, DDT, and SSW). Table 3 presents the T_b_ correlation coefficients as well as *p* values for these comparisons. A Benjamini-Hochberg correction with a false discovery rate of 10% applied to all 35 test comparisons revealed that values of *p* < 0.011 indicated statistically significant correlations. According to this analysis, ΔP1, ΔP2, and ASR habituation were each significantly correlated with behavioral measures. The strongest associations were between ASR habituation and performance on the CWT presented to the left ear (Fig. 5A) and right ear, followed by the association between ΔP2 and CWT presented to the right ear (Fig. 5B). In each case, as predicted, the direction of the correlation indicates that poorer sensory gating is associated with poorer performance on the CWT.

**Table 3.** Correlations between Measures of Sensory Gating and Behavioral Auditory Tests

	*AzBio +5 dB SNR*	*AzBio 0 dB SNR*	*CNC +5 dB SNR*	*DDT*	*SSW*	*CWT Right ear*	*CWT Left ear*
PPI	τb = 0.144	τb = −0.222	τb = −0.027	τb = −0.113	τb = −0.147	τb = 0.135	τb = 0.232
	*p* = 0.392	*p* = .173	*p* = 0.870	*p* = 0.554	*p* = 0.439	*p* = 0.572	*p* = 0.266
Habituation	τb = 0.199	τb = 0.148	τb = 0.211	τb = 0.026	τb = −0.199	**τb = 0.577**	**τb = 0.718**
	*p* = 0.236	*p* = 0.363	*p* = 0.202	*p* = 0.891	*p* = 0.295	***p* = 0.007**	***p* = 0.001**
ΔP1	τb = 0.041	τb = 0.109	τb = 0.058	**τb = 0.408**	τb = −0.281	τb = 0.125	τb = 0.052
	*p* = 0.762	*p* = 0.409	*p* = 0.664	***p* = 0.007**	*p* = 0.064	*p* = 0.455	*p* = 0.756
ΔN1	τb = −0.113	τb = 0.109	τb = −0.043	τb = −0.195	τb = 0.030	τb = −0.031	τb = −0.021
	*p* = 0.405	*p* = 0.409	*p* = 0.784	*p* = 0.200	*p* = 0.841	*p* = 0.852	*p* = 0.901
ΔP2	τb = −0.044	τb = −0.107	τb = 0.061	τb = 0.145	τb = −0.163	**τb = 0.448**	τb = 0.275
	*p* = 0.748	*p* = 0.419	*p* = 0.650	*p* = 0.340	*p* = 0.281	***p* = 0.007**	*p* = 0.100

Bolded values indicated significance at the level of *p* < 0.05.

SNR, signal-to-noise ratio; CNC, Consonant-Nucleus-Consonant; DDT, Dichotic Digits Test; SSW, Staggered Spondaic Words; CWT, Compressed Word Test; PPI, pre-pulse inhibition.

The contribution of demographic variables such as age, TBI diagnosis, PTSD diagnosis, neurobehavioral symptoms, number of brain injuries, time since injury, and years of military service to our sensory gating variables was also assessed using correlations. Poorer ASR habituation was significantly correlated with diagnoses of PTSD (T_b_ = -0.522; *p* = 0.007) and TBI (T_b_ = -0.433; *p* = 0.024), a greater number of total number of brain injuries (T_b_ = -0.435; *p* = 0.012), and with more severe neurobehavioral symptoms (T_b_ = -0.368; *p* = 0.025). Poorer P2 sensory gating (e.g., smaller ΔP2 values) was significantly correlated with diagnoses of TBI (T_b_ = -0.445; *p* = 0.005) and PTSD (T_b_ = -0.405; *p* = 0.010), and with total number of brain injuries (T_b_ = -0.357; *p* = 0.011). No demographic variables were significantly correlated with ΔP1, ΔN1, or PPI.

### Factors predictive of sensory gating and auditory behavioral performance

Because several significant correlations were identified during our initial assessment of the relationship between sensory gating indices and demographic variables, we next performed stepwise linear regression analyses to parse out which factors are most predictive of impaired sensory gating. The effects of TBI diagnosis, PTSD diagnosis, years of military service, and self-report auditory and neurobehavioral symptoms were examined on ΔP2 and on ASR habituation. The probability-of-*F*-to-enter criterion was ≤0.05, and the probability-of-*F*-to-remove criterion was set to ≥0.100 for both analyses. The model predicting habituation to the startle stimulus contained only one predictive variable: diagnosis of PTSD. The model met criteria for statistical significance (*F*_(1,15)_ = 6.796; *p* = 0.021), with PTSD diagnosis accounting for ∼30% of the variance of habituation to the startle stimulus (*R*^2^ = 0.327; adjusted *R*^2^ = 0.279). The model predicting sensory gating indexed by ΔP2 also revealed a single significant predictive variable: diagnosis of TBI. Again, the model met criteria for statistical significance (*F*_(1,22)_ = 13.479; *p* = 0.001), with TBI diagnosis accounting for >35% of the variance of ΔP2 (*R*^2^ = 0.380; adjusted *R*^2^ = 0.352).

**FIG. 6.**
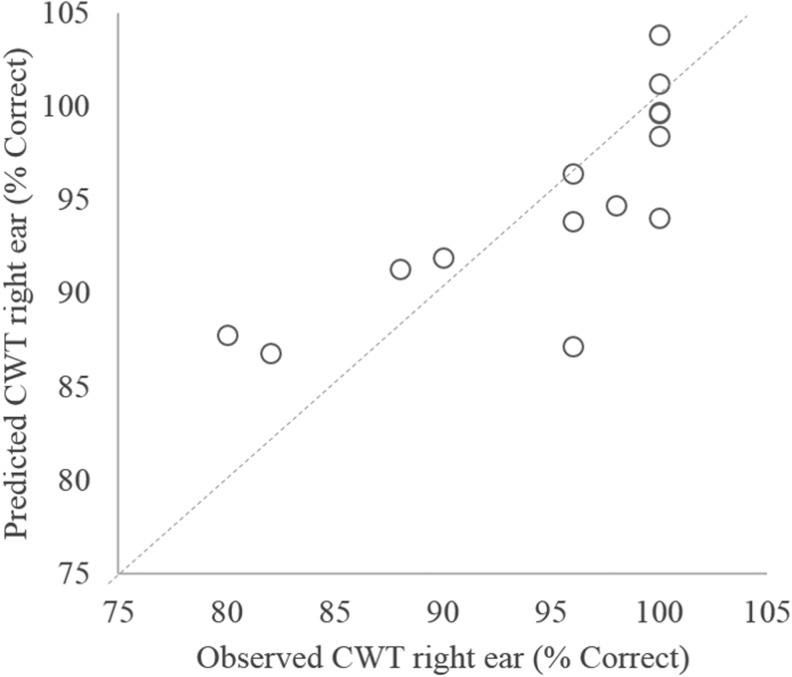
Results of stepwise linear regression exploring the influence of traumatic brain injury (TBI) diagnosis, post-traumatic stress disorder (PTSD) diagnosis, and physiological measures of sensory gating on performance on the Compressed Word Test (CWT) presented to the right ear. Analysis revealed that only ΔP2 and acoustic startle reflex (ASR) habituation were significant predictors of performance.

In addition to investigating the effects of blast exposure on sensory gating, a second question of the current study was whether sensory gating affected performance on auditory tests involving competing or degraded speech. Both ΔP2 and ASR habituation were significantly correlated with the CWT (Table 3; Fig. 5A and B). However, significant correlations with CWT performance were also found for TBI and PTSD diagnoses. To investigate the relative influence of each of these factors on CWT performance, an additional stepwise multiple regression analysis was completed using CWT performance in the right ear as the dependent variable and ΔP2, ASR habituation, PTSD diagnosis, and TBI diagnosis as independent variables. The probability-of-*F*-to-enter criterion was ≤0.05, and the probability-of-*F*-to-remove criterion was set to ≥0.100. The resulting predictive model met criteria for statistical significance (*F*_(2,11)_ = 8.562; *p* = 0.006) and accounted for ∼60% of the variance in CWT performance (*R*^2^ = 0.609; adjusted *R*^2^ = 0.538). ASR habituation alone accounted for 42.7% of the variance in CWT performance, and the addition of ΔP2 accounted for an additional 18.1%. Comparison of the predictive model's performance compared with observed performance values on the CWT are shown in Figure 6.

## Discussion

The present study investigated two primary questions. The first was whether or not exposure to high-intensity blast waves impairs sensory gating. The second was whether or not impaired sensory gating was associated with auditory difficulties. Overall, the results of the present study indicate that blast exposure does increase the risk of sensory gating impairment, as revealed by the significant group differences in both habituation to the acoustic startle stimulus and cortical sensory gating indexed by the P2 peak of the AERP. Sensory gating measures were significantly associated with performance on auditory tasks including dichotic listening, perception of words in noise, and perception of rapid speech. Further, both ASR habituation and ΔP2 were significant predictors of performance on a test of time-compressed speech. These two physiological indicators were better predictors of behavioral auditory performance than any other factors, including diagnoses of TBI, PTSD, age, time in the military, or self-reported measures of hearing abilities.

Currently, the mechanisms through which blast exposure impairs auditory processing are not well understood. The underlying hypothesis of the current study, that sensory gating abnormalities contribute to auditory performance deficits, is based upon the theory that sensory gating plays an important role in the pre-attentive filtering of sensory information for relevant content prior to the processing of this information by higher order cognitive regions.^24,42^ Thus, sensory gating normally helps to ensure that the cortex is not bombarded by extraneous information, and helps to focus higher order cognitive functions such as attention on the most relevant environmental input.^43^ Our data from both ASR habituation and cortical sensory gating generally support this hypothesis.

Although baseline ASR magnitude did not differ between groups, blast-exposed participants failed to habituate to repeated presentations of the startle stimulus, indicating continuing response to repeated, and what should become irrelevant, environmental input. Similarly, although we found no significant difference in the amplitude of response to the conditioning stimulus, P2 responses to the test stimulus (and resulting ΔP2) in the blast-exposed group were significantly larger than responses in the control group, indicating an abnormally large response to redundant stimuli. These findings support the notion that redundant and irrelevant auditory information is often over-represented in the auditory system of veterans who have been exposed to high-intensity blasts.

Impaired sensory gating is likely to affect higher-order functions including speech perception in difficult listening conditions, possibly by diminishing the listener's capacity to attend to multiple acoustic streams simultaneously and apply linguistic analysis in real time. This interpretation is substantiated by factors such as the strong correlations between sensory gating measures and performance on difficult listening tasks (Table 3, Fig. 5), and by the results of other studies indicating that patients with persistent post-concussive symptoms often have mild cognitive impairment and increased response times.^44–46^ Additional physiological evidence comes from multiple studies indicating that the brains of patients with previous blast exposure and mTBI show less distinction between redundant and novel stimuli than neurologically normal brains when measured using AERP oddball stimulus paradigms.^18,21,47,48^ Because sensory gating impairments typically affect more than one sensory modality, it is possible that poor sensory gating contributes to deficits often reported by TBI patients in other sensory domains such as vision and somatosensory stimulation.^12,49–51^

A particularly interesting outcome of the present study was the finding that poor habituation to acoustic startle stimuli was associated with a diagnosis of PTSD, whereas poor cortical sensory gating was associated with a diagnosis of TBI. The relationship among PTSD, TBI, and poor habituation may be explained by the theory of fear conditioning and associated fear-potentiated startle response. Recent work by Callahan and Storzbach^52^ demonstrated a significant relationship between PTSD-related intrusive experiences and noise sensitivity in a deployed veteran sample with blast exposure and mTBI. They found that those who had had trauma-related intrusive experiences maintained a heightened sensitivity to noise consistent with a chronic, maladaptive hyperarousal state.^53,54^ Persistent hyperarousal is associated with excessive autonomic pathway activation,^55,56^ which is in turn associated with increased startle reactivity,^22,57,58^ thus providing a direct autonomic nervous system connection between persistent PTSD symptoms and reduced ASR habituation.^59^

The relationship between reduced P2 sensory gating and TBI diagnosis is likely to be less direct than that between PTSD and poor ASR habituation, because the neural pathway underlying P2 responses is considerably more complex than that controlling the ASR. On the other hand, the complexity of neural underpinnings of P2 responses may be the critical factor linking TBI and P2 sensory gating. Neural damage in TBI is by nature diffuse, heterogenous, and complex. Although a hallmark of mTBI has been a lack of observable structural damage on standard clinical imaging protocols, more advanced imaging techniques as well as animal models have revealed the chronic presence of diffuse axonal injury (DAI) in areas including the temporal and frontal cortices,^60^ interhemispheric tracts including the corpus callosum,^61–64^ cortical-subcortical tracts,^62,63^ frontoparietal tracts,^62^ and cerebellum.^61^ Additional neurobiological effects of TBI include stunted dendritic outgrowths in GABAergic cortical neurons,^65^ mossy fiber sprouting in the hippocampus and increases in excitatory synapses,^66^ and excitatory input to cortical pyramidal neurons.^67^ In the chronic phase of recovery from mTBI, there is considerable evidence of hyperexcitability in sensory cortical areas^65,68,69^ largely driven by decreased activity of inhibitory neurotransmitters and the resulting imbalance of excitatory and inhibitory cortical networks.^70–72^ This pattern of cortical hyperexcitability is likely a consequence of maladaptive restructuring of cortical networks in response to widespread damage.^68,69,73^ AERP P2 response peaks are generated predominately by the primary auditory cortex, which depends on the integrity of the auditory pathway from the cochlea to the cortex, as well as the secondary auditory cortex, which receives input from the primary auditory cortex and from additional cortical and subcortical regions involved in the processing and integration of auditory stimuli.^74,75^ Therefore, the abnormally large P2 responses to redundant stimuli observed in our blast-exposed veterans, and the predictive power of TBI diagnosis on P2 sensory gating, are likely to represent the culmination of diffuse damage to cortical and subcortical neural pathways, and the resulting maladaptive hyperexcitability in the auditory cortex.

Other studies have shown changes in the P1 (also known as P50) amplitude in AERP testing of blast-exposed veterans. Work by Arciniegas and colleagues examined patients who had sustained non-blast-related TBIs at least 1 year prior to testing.^21,76^ Not only were TBI patients found to have reduced P1 sensory gating, but this condition was also found to be associated with reduced hippocampal volume.^77^ Interestingly, the authors reported that P1 response among TBI patients to the conditioning stimulus was significantly smaller than in control participants, whereas the response to the test stimulus was significantly larger. In contrast, we found no significant group differences among P1, N1, or P2 amplitudes in response to the conditioning stimulus (Table 2); only P2 response to the test stimulus was found to be significantly larger in blast-exposed veterans. Discrepancies among studies may be partially the result of methodological differences. For example, the current study employed longer duration stimuli (10 ms vs. 1 ms clicks) presented at a higher intensity, and results were based on responses to an average of 178 trials (minimum of 146), whereas previous studies were based on only 48 stimulus trials with no mention of control for artifacts or eye blinks. Therefore, the AERP results of the current study are undoubtedly less affected by trial-to-trial response variability than those of earlier studies. In addition, differences among participant populations may also explain discrepant study results. The previous studies included individuals with mild, moderate, and severe TBI who also had complaints of persistent cognitive impairments, whereas the current study examined only blast-exposed veterans with a diagnosis of mTBI or who reported symptoms following blast exposure that were consistent with mTBI. Therefore, severity of injury may be a significant factor affecting sensory gating at the thalamocortical levels indexed by the P1 response, whereas sensory gating deficits in those with less severe injuries are more likely to be revealed by early cortical responses. P2 responses have been posited to reflect filtering mechanisms involved in the allocation of attention and working memory^27,78^ whereas P1 responses are believed to reflect the fidelity of stimulus encoding of information from the brainstem to the auditory cortex.^79,80^ Therefore, deficits in P1 and P2 sensory gating likely reflect different filtering mechanisms with potentially different functional outcomes.

### Limitations

Although our study included the use of several complementary methods, including both subjective and objective methods, to ascertain the effect of blast exposure on different levels of auditory function, some limitations remain. First, the sample size was fairly small, and although it was adequately powered to detect significant group differences, it could be susceptible to random sampling effects and sampling bias. For example, the population was primarily self-referred, which inherently carries potential selection bias, although this was mitigated by the use of a self-referred control comparison group. Second, diagnosis of TBI was largely based on self-report and medical record confirmation. Participants were not required to have a medical diagnosis of TBI for study participation both because our previous work has suggested that blast exposure can yield chronic auditory difficulties without a TBI diagnosis, and because many service members report that they are reluctant to report brain injuries to their superiors during active duty in the absence of visible injuries. However, this leaves open the possibility that participants were not accurate in their recollections of symptoms following blast exposure. Lastly, although the findings of associations among TBI, PTSD, and sensory gating are exciting, there was a significant overlap between TBI and PTSD in the current sample. Therefore, these findings should be replicated in future studies that better isolate TBI and PTSD populations.
